# A Case of Traumatic Asphyxia due to Motorcycle Accident

**DOI:** 10.1155/2013/857131

**Published:** 2013-03-31

**Authors:** Sedat Kamali, Sevgi Kesici, Ihsan Gunduz, Ugur Kesici

**Affiliations:** ^1^Department of General Surgery, Okmeydani Training and Research Hospital, Istanbul, Turkey; ^2^Kanuni Training and Reseach Hospital, Department of Anesthesiology and Reanimation, 61290 Trabzon, Turkey; ^3^Department of General Surgery, Akcaabat Hackali Baba State Hospital, Trabzon, Turkey

## Abstract

*Background*. Perthe's syndrome (traumatic asphyxia) is rare, which is caused by sudden compressive chest trauma and characterized by subconjunctival hemorrhage, facial edema, craniocervical cyanosis, and petechiae on the upper chest and face and should always be kept in mind as a possible complication of injuries of the chest and abdomen. *Case Report*. In this case report a 36-years-old male patient brought to the emergency room due to thorax trauma related to motorcycle accident was discussed. Distinct cyanotic, edematous, and multiple petechiae were present on the face, neck, and upper thorax regions of the patient. Bilateral subconjunctival hemorrhage was determined. *Conclusion*. Treatment for traumatic asphyxia is supportive and patient recovery is related to the generally associated injuries. Prognosis of the patients is quite good with effective and timely treatment.

## 1. Introduction

Perthes syndrome (traumatic Asphyxia) is characterized by cyanosis, subconjunctival hemorrhage, and petechiae triad on the head-neck area [1]. First clinically diagnosed traumatic asphyxia cases were reported by Olivier approximately 170 years ago [2]. This syndrome typically arises from sudden and severe trauma of thorax and/or upper abdomen and most cases are caused by motorcycle accidents [3, 4]. Pulmonary contusion, hemothorax, and pneumothorax are the most common injuries accompanying Perthes syndrome [3]. In this case report a male patient with traumatic asphyxia due to motorcycle accident was discussed.

## 2. Case Report

**Figure 1:**
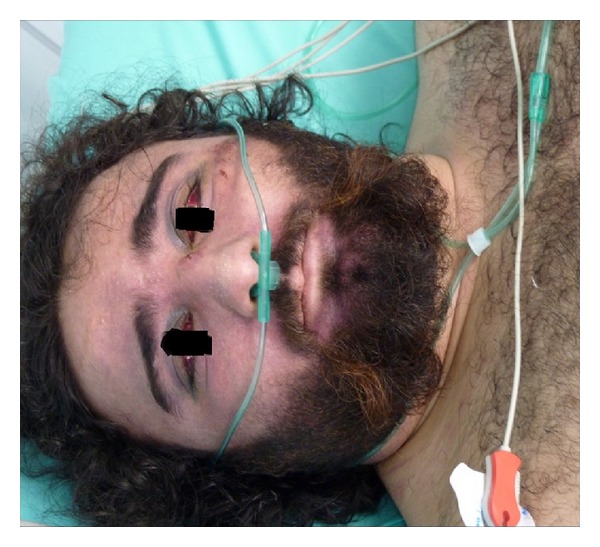
Bilateral subconjunctival hemorrhage.

 In this case report, a 36-years-old male patient who was brought to the emergency room due to thorax trauma related to motorcycle accident was discussed. From the anamnesis of the patient, it was concluded that he got under the bus from one side and while the shaft of the bus rotated, it pulled and crushed the patient between the body of the bus and the shaft and the patient fainted soon afterwards. Direct trauma history could not be obtained from the face and neck area of the patient. He had no features in his history. Pulse was 140/min and tension arterial was 100/65 mm/Hg in the physical examination of the patient. Except these, cardiovascular system examination was normal. Electrocardiogram was monitored as normal. Glasgow Coma Score was 15. Distinct cyanotic, edematous, and multiple petechiae were present on the face, neck, and upper thorax areas of the patient. Bilateral subconjunctival hemorrhage was detected. Subconjunctival hemorrhage of the patient is presented in Figure 1. 

 There was 30 × 40 cm dermabrasion area in the right abdominal region and correspondingly peritoneal irritation findings in the upper right quadrant. Findings like deformation and pathological movement in the left humerus middle 1/3 and left radial nerve paralysis were monitored. Pupillary isochoric and bilateral light reflex were reactive in neurological examination of the patient. Respiratory movements were not detected in the left hemithorax. Distal pulses of both upper extremities were palpable. Increase in aspartate aminotransferase (AST : 2294 U/L) and alanine aminotransferase (ALT : 1902 U/L) was detected in laboratory findings. Hemoglobine count 14.62 g/dL, WBC count 31.390/mm^3^, and platelet count was normal. Arterial blood gas analysis was compatible with metabolic acidosis. Hemoglobin value of the patient dropped inexplicably to 9.05 g/dL during followup of the patient. Two units erythrocyte suspension were replaced. The following day LDH and amylase were measured, respectively, as 3350 U/L 324 U/L. Total bilirubin value was 2.33 mg/dL (maximum) and direct bilirubin value was 0.71 mg/dL. Minimally changed coagulation parameters were recorded as INR 1.23, PT 15.7 sec (52.6%), and aPTT 28.3 sec. Interestingly, creatine kinase value did not increase over 106 U/L.

**Figure 2:**
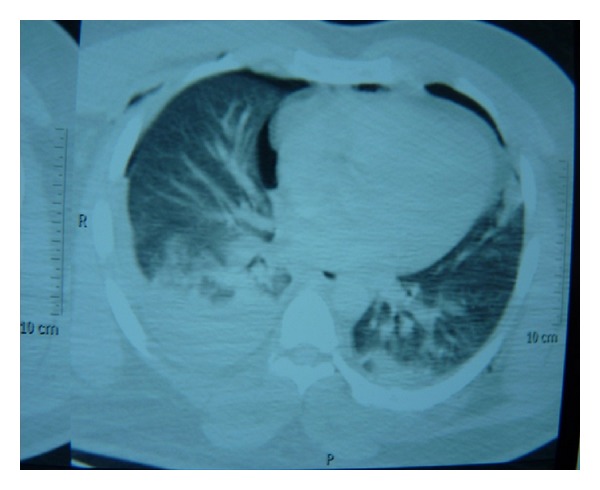
Thorax CT image of the patient.

Patient was quickly evaluated with direct graphics and tomography of brain total spinal, thorax, abdominal-pelvic, and thoracocervical computed tomography (CT) angiography. Displaced fracture was monitored in the left humerus middle 1/3 in direct graphics. In thorax CT, on the right a more apparent and widespread bilateral pulmonary contusion, alveolar hemorrhage, and hemopneumothorax that did not require drainage were detected. Left lateral posterior and left parasternal subcutaneous emphysema were also detected. Fractures were detected in left 7th, 8th, and 9th ribs. Thorax CT image of the patient is presented in Figure 2.

**Figure 3:**
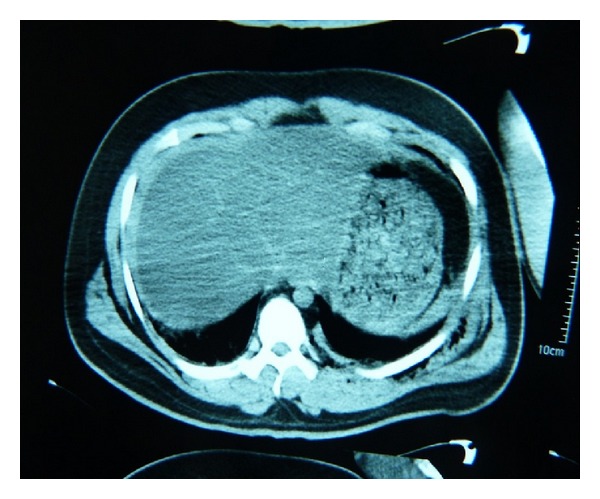
Abdomen CT image of the patient.

Apart from right maxillary and sphenoid hemorrhagic collection, edema and hemorrhage was not detected in brain and spinal tomography. Vascular lesion were not monitored in thorocaservical CT angiography. Perihepatic and perisplenic fluid was detected in abdomen CT. Minimal intrapelvic hemorrhagic fluid was present. Abdomen CT image is presented in Figure 3.

Any abnormal finding was not encountered funduscopically during eye examination. The patient was followed in intensive care unit. Blood gas values in intensive care unit (5 L/min, under oxygen with 100% mask) were pH 7.30, PaCO_2_ 45 mmHg, PaO_2_ 60 mmHg, and base excess −2.8 mMol · L^−1^. Intravenous bolus of methylprednisolone (30 mg/kg), methylprednisolone infusion of 5.4 mg/kg per hour for 24 hours, fluid replacement, and oxygen support with mask were performed to the patient. Reduction and splint were performed for the fracture in left humerus. Mechanical ventilation was not necessary during followup. No complications arose during treatment. Length of hospital stay was 7 days.

## 3. Discussion

Although traumatic asphyxia cases were first reported by Olivier with craniofasial cyanosis, subconjunctival hemorrhage and cerebrovascular congestion findings in autopsied peoples and subsequently similar cases were reported in the literature in France and Germany, and no definition was made. In 1900, Perthes observed cases diagnosed with mental confusion, hyperpyrexia, hemoptysis, tachypnea, and contusion pneumonia and cases diagnosed subsequently with progressing petechial bleedings in mucosal membranes, epistaxis, hematemesis, rectal bleeding, esophageal, hematoma, albuminuria, microscopic hematuria, paraplegia, peripheral nerve damage, amnesia, and convulsion were monitored, and for the first time it was described with presently known term “Traumatic Asphyxia” [5].

 Traumatic asphyxia is a rare condition presenting with cervicofacial cyanosis and edema, petechial, and subconjunctival hemorrhages of the face, neck, and upper chest that occurs usually due to a compressive force to the thoracoabdominal region but has also been associated with asthma, paroxysmal coughing, protracted vomiting, and jugular venous occlusion [6, 7]. Factors implicated in the development of these striking physical characteristics include thoracoabdominal compression after deep inspiration against a closed glottis, which results in venous hypertension in the valveless cervicofacial venous system [8]. Alternatively, increased airway pressure may compress or obliterate the inferior vena cava to protect the lower part of the body [9]. In the literature it is reported that traumatic asphyxia might develop during 2–5 minutes (average) compression period [4]. Although most cases are seen as a result of motorcycle accident other causes include heavy machines/furniture crashes and rarely, deep-sea diving, epileptic seizures, difficult delivery, and asthmatic attack [2]. Senoglu et al. [4] present a 4-year-old child developing traumatic asphyxia associated with intramedullary spinal cord hemorrhage fallowed by thoracal compression. They said that it is important to the clinicians to be aware of the spinal cord hemorrhage that can be accompanied to the traumatic asphyxia and treated with steroid immediately after the trauma without radiological evidence. 

Problems relating to other organ injuries can clinically be accompanied in patients with traumatic asphyxia [10]. The study performed by Jongewaard et al. [11] reported 2 cases of visual impairment, 2 cases of epileptic seizure, 5 cases of prolonged confusion, 8 cases of loss of consciousness, and 11 cases of chest wall and intrathoracic injury in 14 patients with Perthes syndrome. In our case, generalized bilateral pulmonary contusion that is more distinct in the right alveolar hemorrhage and hemopneuomthorax not requiring drainage were detected. Although Perthes syndrome is seen rarely in the clinics, in the paper by Olusina et al. they stated that as a result of autopsy performed to 10 out of 14 people who died during a social event, in 60% traumatic asphyxia was detected. 

 Traumatic asphyxia has a good prognosis. Supportive treatment such as oxygenation and elevation of the head to 30° is usually sufficient in the management of these patients. However, specific treatments may be needed for the associated injuries [12]. In our case, supportive therapy and specific treatment for left humerus fracture were performed. Additional surgical treatment was not required for other organ pathologies and no complication arose during followups.

 In conclusion, when characteristic findings of traumatic asphyxia are detected in traumatic patients, other organ pathologies should be quickly eliminated and supportive therapy should be initiated. If any other organ pathology is detected, treatment for the detected pathology should be administered because the prognosis of patients with timely and effective treatment is considerably good.
